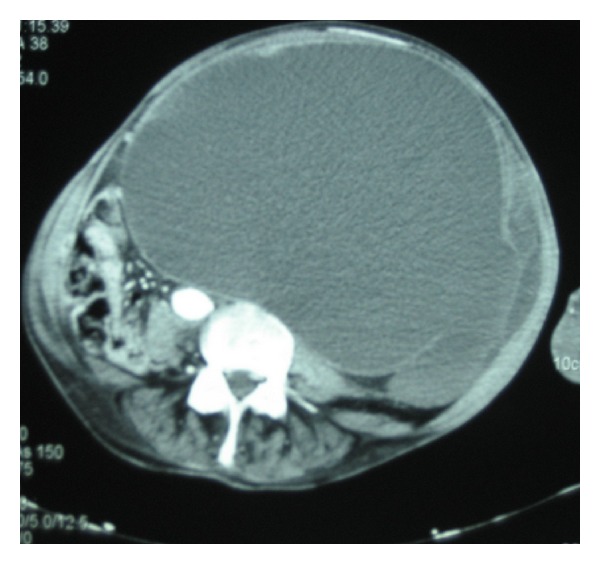# About a Case Report of Giant Hydronephrosis

**DOI:** 10.1155/2013/257969

**Published:** 2013-09-29

**Authors:** Enrique Mediavilla, Roberto Ballestero, Miguel Angel Correas, Jose Luis Gutierrez

**Affiliations:** Hospital Universitario Marqués de Valdecilla, Facultad de Medicina, Universidad de Cantabria, Servicio de Urología, Avenida Valdecilla No. 25, Santander, 39008 Cantabria, Spain

## Abstract

*Introduction*. Our objective is to report a case of an infrequent entity as the giant hydronephrosis. *Case Report*. We report the case of an 82-syear-old male referred for a poor general condition. A radiological study revealed a great left hydronephrosis secondary to an urothelial carcinoma. The patient died due to his poor general condition. A histological diagnosis revealed a transitional cell carcinoma of renal pelvis and ureter and atrophic renal parenchyma. *Conclusion*. Giant hydronephrosis represents a very often entity to be taken into account in cases with big cystic abdominal masses in absence of unilateral or bilateral kidney. Simple nephrectomy is the treatment of choice in most cases. Nevertheless, in cases of nonsubsidiary surgery, percutaneous drainage may be necessary.

## 1. Introduction

Since Stirling defined giant hydronephrosis as the amount of fluid exceeding 1,000 cc in the urinary tract of adults, very few cases have been reported in adults, because it is very difficult to diagnose due to the lack of a well-defined pattern. The aim of this paper is to present one case of hydronephrosis along with a review of the current literature.

## 2. Clinical Case

An 82-year-old male was brought to the emergency room for a serious deterioration of his general condition with abdominal pain and an abdominal mass effect. It was impossible to obtain information about the patient's clinical history due to his lack of cooperation and lack of information from his companion. 

Upon physical examination, the patient showed symptoms of a stupor state without focal neurologic signs, severe malnutrition, dehydration (positive skin pinch), and a tense abdomen with a large abdominal mass, which appeared to be located on the left hemiabdomen. No signs of peritonitis were observed.

**Figure 1:**
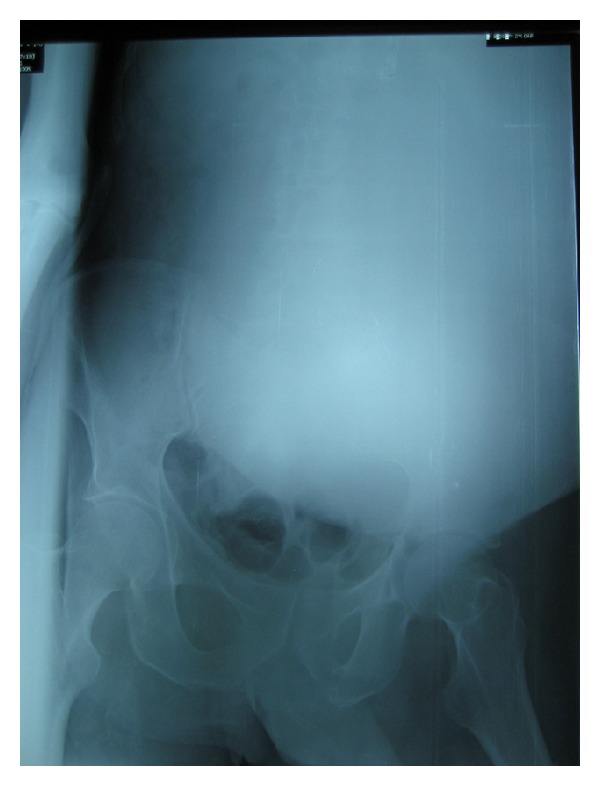


The blood laboratory tests highlighted a haematocrit of 14.3%, haemoglobin of 4.0 g/dL, creatinine of 2.1 mg/dL, and urea of 89 mg/dL. The abdominal radiograph (Figure 1) showed a large abdominal mass of water density in the left hemiabdomen.

A complementary radiological study via abdominal ultrasound and CT scan (Figure 2) was performed and showed a large cystic mass (22.5 × 16.5 cm), which occupied the abdominal cavity and displaced the abdominopelvic structures. The mass appeared to extend up to the bladder and increased in density near the union; however, the origin of the union could not be determined by the aforementioned imaging techniques. Nodular images of higher density were observed within the mass and may have corresponded to clots. The left renal parenchyma was not observed. 

Upon admission, we punctured the cystic mass and removed 5,000 cc of serosanguineous fluid. The biochemical analysis of the fluid revealed the levels of 6 mg/dL for glucose, 1.5 mg/dL for creatinine, 127 mg/dL for urea, 137 mEq/L for sodium, and 4 mEq/L for potassium. 

The patient progressed unfavourably during admission and died two days later despite fluid resuscitation and blood transfusions.

During necropsy, the abdominal mass was reported as a left renal mass weighing 4.500 g, with dimensions of 30 × 21 × 10 cm, resulting from ureterohydronephrosis. The histopathology sample showed a multifocal, extensively necrotic, and WHO Grade 2 papillary transitional cell carcinoma with invasion of the superficial muscular layer (pT2a), which affected the renal pelvis and the left ureter up to the bladder. Remnants of atrophic renal parenchyma were observed.

Thromboembolism of the left main pulmonary artery was determined as the cause of death. 

We present this case to show the final evolution of the urothelial carcinoma using images. 

## 3. Discussion

Giant hydronephrosis is a condition caused by the accumulation of more than 1,000 mL in the excretory system of either kidney, as defined by Stirling in 1939 [1–4]. The first case was published in 1746, and more than 600 cases have been described worldwide to date, with most cases reported within the last 15 years [1, 4].

The most common cause of giant hydronephrosis, as described in the literature, is congenital stenosis of the ureteropelvic junction associated with other urinary tract abnormalities; other causes include urolithiasis and tumour pathology [1, 4] of the urinary tract (as in our case) or the nearby organs, which may cause compression of the kidney.

Epidemiologically, giant hydronephrosis is more common in the left kidney of males with an average content of 1-2 L in the collecting system [1].

The clinical symptoms of these patients are not specific but typically involve increased abdominal girth due to the presence of a mass in the flank. Other symptoms were described in the literature, including pain at the flank along with haematuria resulting from trauma in the area [4].

Historically, only 50% of giant hydronephrosis cases are properly diagnosed because of its nonspecific clinical presentation [4, 5]. However, imaging techniques have been improved dramatically in recent years. Using ultrasonography, giant hydronephrosis can be defined as the presence of hydronephrosis extending beyond the abdominal midline or extending to five or six vertebral bodies without renal parenchyma in radiological features from that area [4]. Other useful diagnostic imaging techniques include abdominal radiography with observable radiopaque lithiasis, intravenous urography (IVU) showing no excretion in the affected kidney, and CT. Curiously, we observed an increase of CA 19-9 in cases of ureteral lithiases [3].

The following differential diagnoses were considered: massive ascites [5, 6], intraperitoneal cysts, renal or adrenal retroperitoneal cysts, pancreatic pseudocysts, and ovarian cysts or tumours [4].

The ideal treatment for giant hydronephrosis is simple nephrectomy, as proposed by Hoffman, because of the frequent association of foci of dysplasia and tumour changes of the parenchyma and collecting system of kidneys as a result of chronic irritation [3]. However, a puncture/drain procedure may also be performed in cases in which the condition of the patient does not allow other treatments or when hemodynamic changes can occur following a sudden abdominal decompression [1, 2].

In our patient, giant hydronephrosis was caused by terminal-stage, upper urinary tract transitional cell carcinoma, which is particularly uncommon.

In conclusion, giant hydronephrosis is a rare condition that must be considered upon the occurrence of cystic abdominal masses and the absence of one or both kidneys. Although its aetiology is diverse, late-stage tumour pathologies such as urothelial carcinoma must be considered as potential causes of giant hydronephrosis.

## Figures and Tables

**Figure 2:**